# Hematopoietic cytoplasmic adaptor protein Hem1 promotes osteoclast fusion and bone resorption in mice

**DOI:** 10.1016/j.jbc.2022.102841

**Published:** 2022-12-24

**Authors:** Xiaoyan Wang, Lijian Shao, Kimberly K. Richardson, Wen Ling, Aaron Warren, Kimberly Krager, Nukhet Aykin-Burns, Robert Hromas, Daohong Zhou, Maria Almeida, Ha-Neui Kim

**Affiliations:** 1 Department of Pharmaceutical Sciences and Winthrop P. Rockefeller Cancer Institute, University of Arkansas for Medical Sciences, Little Rock, Arkansas, USA; 2 Division of Endocrinology, Department of Internal Medicine, Center for Musculoskeletal Disease Research and Center for Osteoporosis and Metabolic Bone Diseases, University of Arkansas for Medical Sciences, Little Rock, Arkansas, USA; 3 Central Arkansas Veterans Healthcare System, Little Rock, Arkansas, USA; 4 Division of Radiation Health, Department of Pharmaceutical Sciences, University of Arkansas for Medical Sciences, Little Rock, Arkansas, USA; 5 Department of Medicine, The Long School of Medicine, University of Texas Health Science Center at San Antonio, San Antonio, Texas, USA; 6 Department of Pharmacodynamics, University of Florida, Gainesville, Florida, USA; 7 Department of Orthopedic Surgery, University of Arkansas for Medical Sciences, Little Rock, Arkansas, USA

**Keywords:** Hem1, osteoclasts, osteoblasts, fusion, bone resorption, bone formation, hematopoiesis, cDNA, complementary DNA, CFU, colony-forming unit, CFU-AD, CFU adipocyte, CFU-F, CFU fibroblast, CFU-OB, CFU-osteoblast, FBS, fetal bovine serum, Hem1, hematopoietic protein 1, MAPK, mitogen-activated protein kinase, M-CSF, macrophage colony–stimulating factor, RANKL, receptor activator of nuclear factor-kappa B ligand, TRAP, tartrate-resistant acid phosphatase, WAVE, WASP (Wiskott–Aldrich syndrome protein)-family verprolin homologous protein

## Abstract

Hem1 (hematopoietic protein 1), a hematopoietic cell–specific member of the Hem family of cytoplasmic adaptor proteins, is essential for lymphopoiesis and innate immunity as well as for the transition of hematopoiesis from the fetal liver to the bone marrow. However, the role of Hem1 in bone cell differentiation and bone remodeling is unknown. Here, we show that deletion of Hem1 resulted in a markedly increase in bone mass because of defective bone resorption in mice of both sexes. Hem1-deficient osteoclast progenitors were able to differentiate into osteoclasts, but the osteoclasts exhibited impaired osteoclast fusion and decreased bone-resorption activity, potentially because of decreased mitogen-activated protein kinase and tyrosine kinase c-Abl activity. Transplantation of bone marrow hematopoietic stem and progenitor cells from wildtype into *Hem1* knockout mice increased bone resorption and normalized bone mass. These findings indicate that Hem1 plays a pivotal role in the maintenance of normal bone mass.

Bone is a highly dynamic tissue that responds and adapts to changes in systemic signals and to mechanical forces. Bones regenerate periodically in discrete sites *via* a remodeling process through which old or damaged bone is resorbed by osteoclasts and is replaced with new bone by osteoblasts (1, 2).

Osteoclasts differentiate from hematopoietic precursor cells of the monocyte/macrophage lineage in response to two critical osteoclastogenic cytokines—macrophage colony–stimulating factor (M-CSF) and receptor activator of nuclear factor-kappa B ligand (RANKL) (3). Osteoclasts develop in several steps, beginning with proliferation of hematopoietic progenitor cells that differentiate to mononuclear preosteoclasts that then fuse to become multinucleated mature osteoclasts (3). These cells are uniquely capable of dissolving and digesting the bone matrix because of their ability to trigger actin polymerization in actin ring and to form a “podosome belt” that tightly adheres to the bone area that is targeted for removal, creating a sealed microenvironment into which the cells secrete protons and lysosomal enzymes (3, 4). The high energy demands of these tasks are likely the reason for the abundance of mitochondria within osteoclasts, a distinct cellular feature of these cells (5, 6). The balancing act of healthy bone remodeling is accomplished by osteoblasts refilling each resorption cavity with new bone. Osteoblasts derive from mesenchymal stem cells present in the bone marrow (1). Osteoblast differentiation occurs in response to osteoblast-formation signals, including matrix-derived factors released during bone resorption. The contribution of osteoclast-derived signals or factors released during resorption to osteoblast generation is referred to as “coupling” (7, 8).

Hematopoietic protein 1 (Hem1), also known as Nck-associated protein 1-like (NAP1l or Nckap1l), is a member of the Hem family of cytoplasmic adaptor proteins. Orthologs of Hem1 in lower organisms, such as *Drosophila melanogaster* and *Caenorhabditis elegans*, are essential for cytoskeletal reorganization, embryonic cell migration, and morphogenesis (9, 10, 11). Studies in mice and cell lines indicate that Hem1 is a component of the “WAVE (WASP [Wiskott–Aldrich syndrome protein]-family verprolin homologous protein)” complex, which signals downstream of activated Rac and upstream of the Arp2/3 complex to stimulate actin polymerization in response to immunoreceptor signaling (9, 12). Recent work from our group has shown that mice with deletion of Hem1 exhibit growth retardation and premature death at about 6 weeks. These defects were associated with premature exhaustion of neonatal bone marrow hematopoietic stem cells, indicating that Hem1 is required for hematopoiesis to transition from the fetal liver to the bone marrow (13). In contrast, Hem-1 is essential for the normal development and function of the other organ systems. Because osteoclasts differentiate from the hematopoietic lineage, we examined the roles played by Hem1 in the skeleton. Here, we show that deletion of Hem1 led to a defect in osteoclast maturation, a decrease in bone resorption, and a marked increase in trabecular bone volume. All these abnormalities could be corrected by transplantation of normal bone marrow hematopoietic stem and progenitor cells.

## Results

### Hem1 knockout mice have increased bone mass

**Figure 1:**
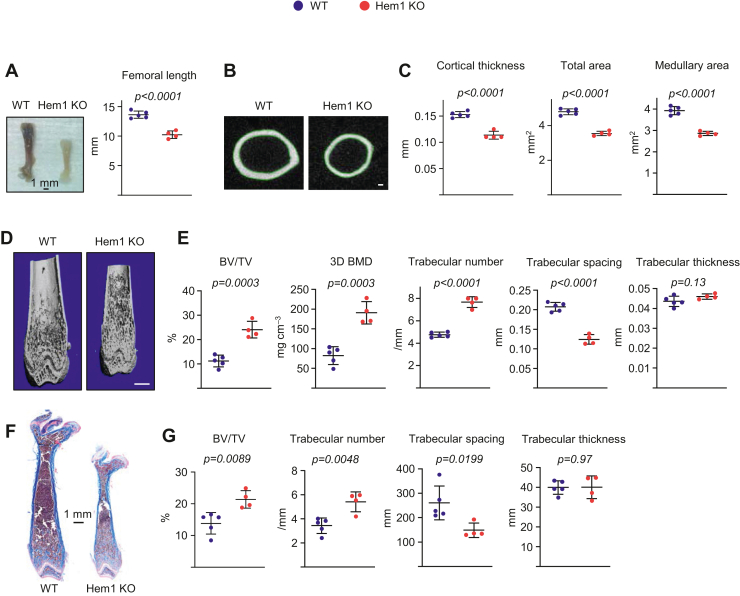
**Deletion of Hem1 increases trabecular bone mass in mice.** Micro-CT imaging and quantification of femoral bones from male Hem1 knockout mice and wildtype littermates at 5.5 weeks of age. *A*, representative images of whole femur (*left*) and length measurement (*right*). *B*, representative images of cortical bone at midshaft (scale bar represents 100 μm). *C*, cortical thickness and cortical perimeter at midshaft. *D*, representative images of trabecular bone (scale bar represents 1 mm) and (*E*) bone volume per tissue volume (BV/TV), bone mineral density (BMD), and microarchitecture of trabecular bone. *F*, histological sections of distal femurs stained with Masson's trichrome. *G*, standard histomorphometric parameters quantifying trabecular architecture (BV/TV, trabecular number, thickness, and spacing) in histologic sections of 5.5-week-old male mice. *Lines* and error bars represent mean ± SD; n = 4 to 5 animals/group. *p* Values were determined with Student’s *t* test. Hem1, hematopoietic protein 1.

To address the role of Hem1 in bone remodeling, we generated Hem1 knockout mice as described previously (13). At 5.5 weeks of age, Hem1 knockout mice had reduced body size, body weight (13), and femoral length than their littermate wildtype controls (Fig. 1*A*). The morphology of the femoral growth plate was not overtly affected indicating that the defects in bone size do not seem to result from defective growth plate chondrogenesis (Fig. S1). Consistent with the reduced femoral length in Hem1 knockout mice, the femurs had lower cortical thickness, total area, and medullary area than wildtype littermates (Fig. 1, *B* and *C*), as determined by micro-CT. In contrast, distal femurs of both male and female Hem1 knockout mice had elevated trabecular bone volume and bone mineral density (Figs. 1, *D* and *E* and S2) because of an increase in trabecular number and a decrease in trabecular spacing. Trabecular thickness was unchanged in male (Fig. 1*E*) but slightly increased in female Hem1 knockout mice (Fig. S2). Consistent with the micro-CT results, histological analysis of femoral bone showed higher trabecular bone area and trabecular number and reduced trabecular spacing in Hem1 knockout mice than in wildtype controls (Fig. 1, *F* and *G*).

### Hem1 knockout mice have more osteoclasts but less bone resorption than wildtype mice

**Figure 2:**
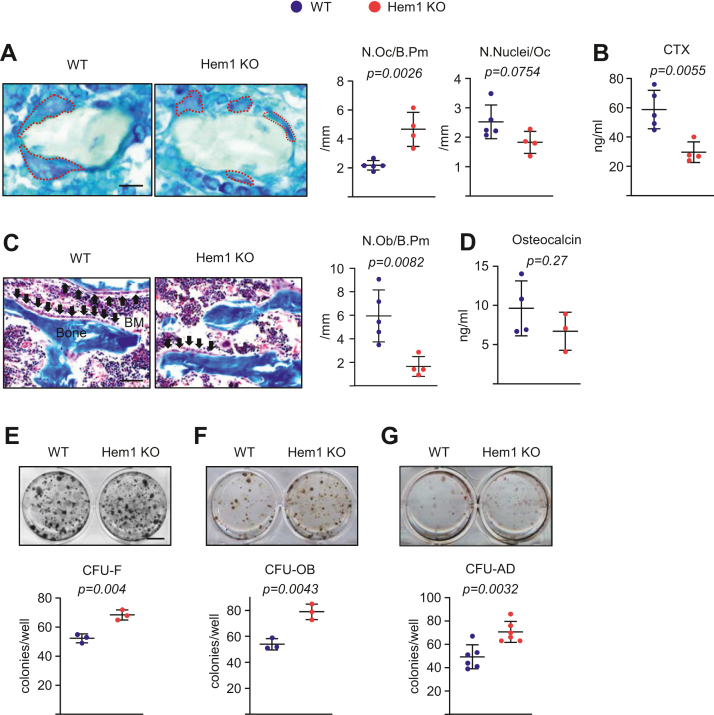
**Deletion of Hem1 in mice decreases bone resorption.***A*, representative photomicrographs of osteoclasts (*left*), number of osteoclasts (*middle*; N.Oc/B.Pm), and nuclei number per osteoclast (*right*; N.Nuclei/Oc) per trabecular bone surface of nondecalcified femur sections stained for TRAP activity (*red arrows*) from 5.5-week-old male Hem1 knockout mice and wildtype littermates (n = 4–5 animals/group) (scale bar represents 400 μm). *Dotted red lines* indicate multinucleated TRAP-positive osteoclasts. *B*, ELISA analysis of serum concentration of a collagen degradation product (CTx) in 5.5-week-old male Hem1 knockout mice and wildtype littermates (n = 4–5 animals/group). *C*, representative photomicrographs of osteoblasts (*left*; *black arrows*) and number of osteoblasts (*right*; N.Ob/B.Pm) per trabecular bone surface of nondecalcified femur sections stained for Masson's trichrome from 5.5-week-old male Hem1 knockout mice and wildtype littermates (n = 4–5 animals/group) (scale bar represents 100 μm). *D*, ELISA analysis of serum concentration of osteocalcin (n = 3–4 animals/group). *E*–*G*, photomicrographs show representative colonies (*top*). CFU-F stained for alkaline phosphatase after 10 days, CFU-AD stained with Oil Red O after 7 days, and CFU-OB stained with von Kossa after 25 days to detect mineral (*bottom*) (triplicate cultures) (scale bar represents 1 cm). *Lines* and error bars represent mean ± SD. *p* Values were determined with Student’s *t* test. CFU-AD, CFU adipocyte; CFU-F, CFU fibroblast; CFU-OB, CFU-osteoblast; Hem1, hematopoietic protein 1; TRAP, tartrate-resistant acid phosphatase.

To determine the cellular basis of the increase in trabecular bone mass, we enumerated osteoclasts and osteoblasts in the distal femur. Surprisingly, the knockout mice had increased numbers of osteoclasts, as determined by tartrate-resistant acid phosphatase (TRAP) staining in histological sections (Fig. 2*A*). These changes were associated with an increase in the number of myeloid cells in the bone marrow (Fig. S3). However, bone resorption as determined by serum levels of collagen degradation product (C-terminal telopeptide of type 1 collagen (CTx)) was severely reduced from that seen in wildtype controls (Fig. 2*B*). A decrease in osteoclast multinucleation has been associated with decreased bone resorption (14). In an attempt to explain the discrepancy between osteoclast number and bone resorption in Hem1 knockout mice, we quantified the number of nuclei in osteoclasts. Hem1 knockout mice exhibited lower number of nuclei per osteoclast than wildtype mice (Fig. 2*A*).

Consistent with the observed reduction in bone resorption, osteoblast numbers were lower in Hem1 knockout mice (Fig. 2*C*). The serum levels of the bone formation marker osteocalcin were not impacted in Hem1 knockout mice (Fig. 2*D*). This is most likely because of the increase in trabecular bone mass and consequently an increase in bone surfaces, which could compensate for the decrease in osteoblast number per bone surface. To examine whether the number of mesenchymal progenitors was affected by Hem1 deletion, we quantified the number of colony-forming unit (CFU)-fibroblasts (CFU-Fs), CFU-osteoblasts (CFU-OBs), and CFU-adipocytes (CFU-ADs) formed by mesenchymal progenitors from wildtype and Hem1 knockout mice in *ex vivo* bone marrow cultures. The numbers of CFU-F, CFU-OB, and CFU-AD were greater in Hem1 knockout mice than in wildtype mice (Fig. 2, *E*–*G*). In addition, we counted CD45−Lin−CD31−Sca1+CD51+ mesenchymal stem cells in the bone marrow and found that, consistent with the observed increase in number of CFU, the number of this population was greater in Hem1 knockout mice (Fig. S4*A*). Strikingly, however, osteoblast and adipocyte differentiation as determined by mRNA levels of *ALP* and *PPARγ*, respectively, were reduced from that seen in wildtype littermate controls (Fig. S4*B*). We next performed high-density cultures of bone marrow stromal cells to examine possible effects on osteoblast differentiation. Cells from Hem1 knockout mice exhibited lower osteoblast maker levels (*i.e.*, *Runx2*, *Osterix*, and *Osteocalcin*) when cultured under osteogenic conditions (Fig. S4*C*). These results indicate that the low osteoblast number in knockout mice is not because of reduced mesenchymal progenitors. However, these progenitors seem to be less differentiated in the absence of Hem1. In any case, even if a decrease in osteoblast differentiation contributes to the decrease in the number of osteoblasts, these events could not explain the high bone mass in Hem1 knockout mice. Taken together, these findings suggest that the high bone mass of Hem1 knockout mice is due to a decrease in osteoclastic bone resorption.

### Deletion of Hem1 decreases osteoclast fusion and activity *in vitro*

**Figure 3:**
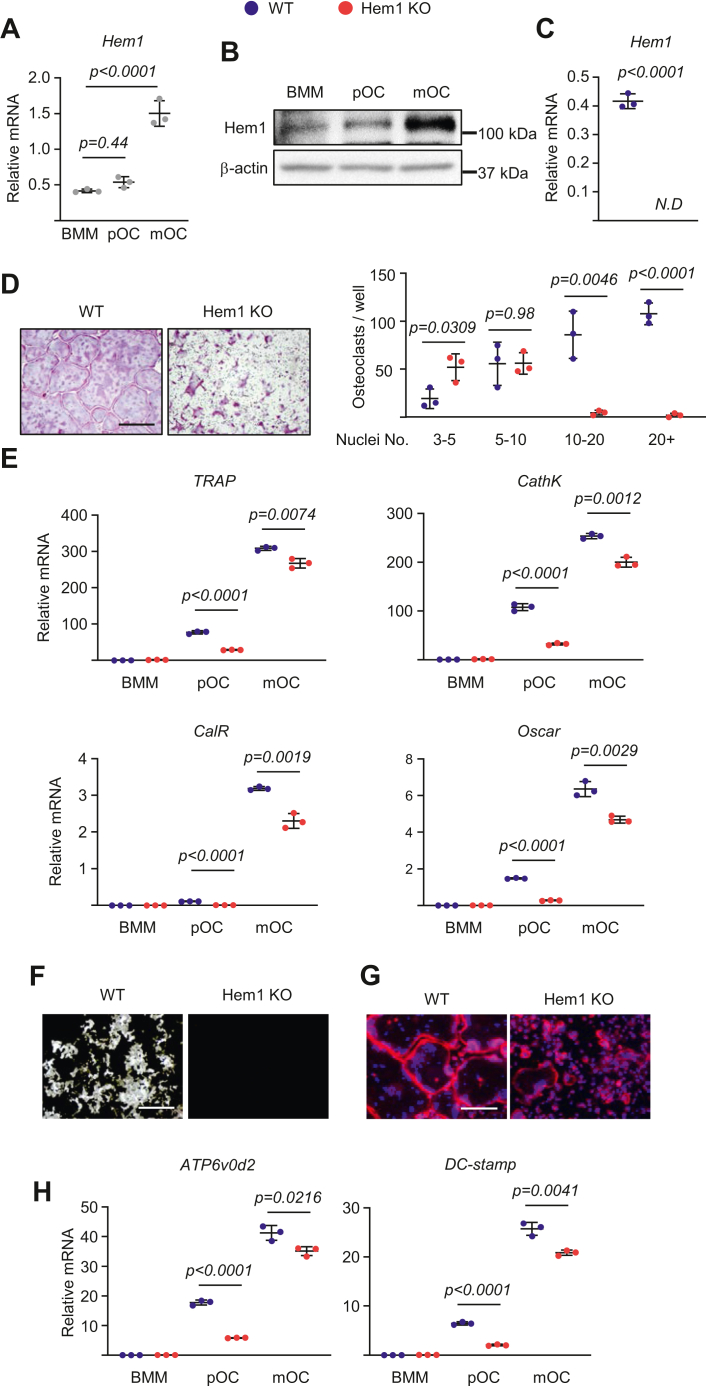
**Deletion of Hem1 decreases osteoclast fusion.***A* and *B*, bone marrow macrophages were isolated from 6-month-old C57BL/6 wildtype mice and were cultured with M-CSF (30 ng ml^−1^, bone marrow macrophages) or with M-CSF and RANKL (30 ng ml^−1^) for 2 days (pOC) or 5 days (mOC). Levels of Hem1 (*A*) mRNA (quantitative PCR [qPCR] assay) and (*B*) protein (Western blot) during osteoclastogenesis. *C*–*H*, bone marrow macrophages were isolated from 5.5-week-old male Hem1 knockout mice and wildtype littermates and were cultured with M-CSF (30 ng ml^−1^) and RANKL (30 ng ml^−1^) for (*C*) 24 h, (*D*, *F*, and G) 5 days, (*H*) 3 days, or (*E* and *H*) indicated periods. *C*, Hem1 mRNA levels (qPCR assay) during osteoclastogenesis. *D*, representative pictures (*left*) and number (*right*) of TRAP-positive multinucleated osteoclasts generated from bone marrow macrophages (scale bar represents 500 μm). *E*, mRNA levels (qPCR assay) of osteoclast markers during osteoclastogenesis. *F*, representative pictures of Von Kossa-stained bone biomaterial surface (scale bar represents 500 μm) (n = 4/group). The resorbed areas appear *white*, and the unresorbed mineralized surface appears *black*. *G*, representative pictures of DAPI (*blue*; nuclei) and phalloidin (*red*; actin rings) staining of osteoclast cultures (scale bar represents 500 μm). *H*, mRNA levels (qPCR assay) of osteoclast fusion markers during osteoclastogenesis. All cultures were completed in triplicate. *Lines* and error bars represent mean ± SD. *p* Values were determined with (*A*) one-way ANOVA or (*C*–*H*) Student’s *t* test. DAPI, 4′,6-diamidino-2-phenylindole; Hem1, hematopoietic protein 1; M-CSF, hematopoietic protein 1; RANKL, receptor activator of nuclear factor-kappa B ligand; TRAP, tartrate-resistant acid phosphatase.

To investigate the role of Hem1 in osteoclastogenesis, bone marrow macrophages were cultured in the presence of M-CSF and RANKL. The *Hem1* mRNA expression and protein levels was about threefold higher in mature osteoclast than macrophages or preosteoclasts from wildtype mice (Fig. 3, *A* and *B*). Macrophages from Hem1 knockout mice failed to form multinucleated giant osteoclasts (with more than ten nuclei) but formed a higher number of smaller osteoclasts (with 3–5 nuclei) than those from wildtype mice (Fig. 3, *C* and *D*). In agreement with these findings, the levels of mature osteoclast markers (*TRAP*, *cathepsin K*, *calcitonin receptor*, and *Oscar*) were lower in cultures from Hem1-deficient mice than in those from wildtype littermates (Fig. 3*E*).

The findings that bone resorption is greatly decreased in Hem1 knockout mice associated with deficient osteoclast multinucleation led us to focus our attention on the impact of Hem1 on osteoclast resorptive capacity. To this end, osteoclasts from wildtype and Hem1 knockout mice were cultured on plates coated with a bone biomimetic synthetic surface. We found that the area resorbed (Fig. 3*F*) and actin ring formation (Fig. 3*G*) by osteoclasts of Hem1-deficient mice were greatly diminished compared with the cells from wildtype mice. Consistent with these results, mRNA levels of Dc-stamp and Atp6v0d2, which are involved in osteoclast fusion and actin ring formation (15, 16), were significantly decreased in preosteoclasts and osteoclasts from Hem1-deficient mice (Fig. 3*H*).

**Figure 4:**
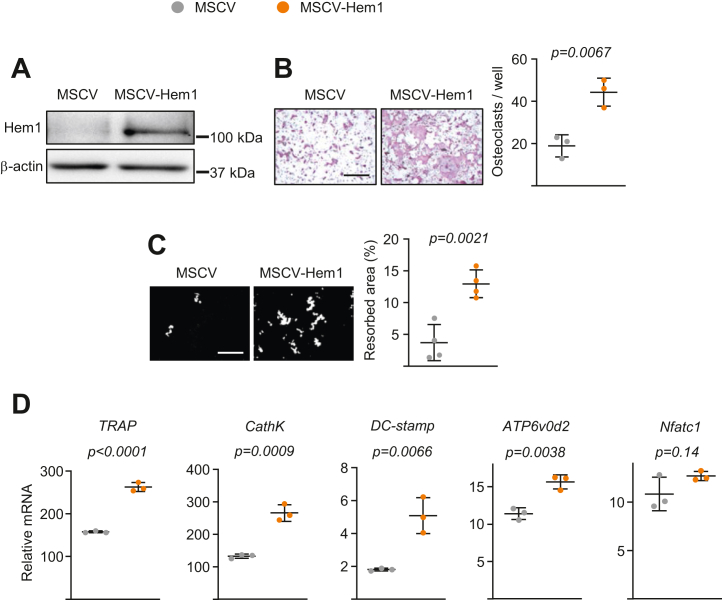
**Rescue of cell fusion in Hem1-deficient osteoclasts by retroviral transduction of Hem1.** Bone marrow macrophages lacking Hem1 were transduced with retroviral vectors expressing either empty vector (MSCV) or Hem1 (MSCV-Hem1) and were cultured with M-CSF and RANKL for (*A*) 24 h or (*B*–*D*) 5 days. *A*, protein levels (Western blot) of Hem1 in bone marrow macrophage cell cultures. *B*, representative pictures (*left*) and number (*right*) of TRAP-positive multinucleated osteoclasts with more than ten nuclei, generated from bone marrow macrophages (scale bar represents 500 μm). *C*, representative pictures (*left*) and resorbed areas (*right*) of Von Kossa-stained bone biomaterial surface (scale bar represents 500 μm) (n = 4/group). *D*, mRNA levels (quantitative PCR assay) of osteoclast markers in mOCs. All cultures were completed in triplicate. *Lines* and error bars represent mean ± SD. *p* Values were determined using Student’s *t* test. Hem1, hematopoietic protein 1; M-CSF, macrophage colony–stimulating factor; MSCV, murine stem cell virus; RANKL, receptor activator of nuclear factor-kappa B ligand; TRAP, tartrate-resistant acid phosphatase.

To confirm that the defects seen in bone resorption of Hem1 knockout mice were due to direct effects of Hem1 in macrophages, we expressed Hem1 in bone marrow macrophage cultures from Hem1 knockout mice (Fig. 4*A*). Expression of Hem1 increased osteoclast formation (Fig. 4*B*) and resorption capacity by twofold to threefold (Fig. 4*C*). The mRNA levels of osteoclast-related genes (*TRAP*, *Cathepsin K*, *Dc-stamp*, and *Atp6v0d2*) also increased in response to Hem1 expression (Fig. 4*D*).

Cells of the mesenchymal lineage including stromal cells are major producers of RANKL and other cytokines that support osteoclastogenesis. We also examined whether osteoclast differentiation in Hem1 knockout mice could be affected indirectly by bone marrow–mesenchymal cells. To this end, we cocultured bone marrow macrophages from wildtype mice with stromal cells from wildtype or Hem1 knockout mice. Stromal cells from either genotype equally supported osteoclast formation (Fig. S5). These data further support to the idea that Hem1 contributes directly to the formation of multinucleated osteoclasts.

### Deletion of Hem1 decreases mitochondrial respiration and c-Abl signaling in osteoclasts

**Figure 5:**
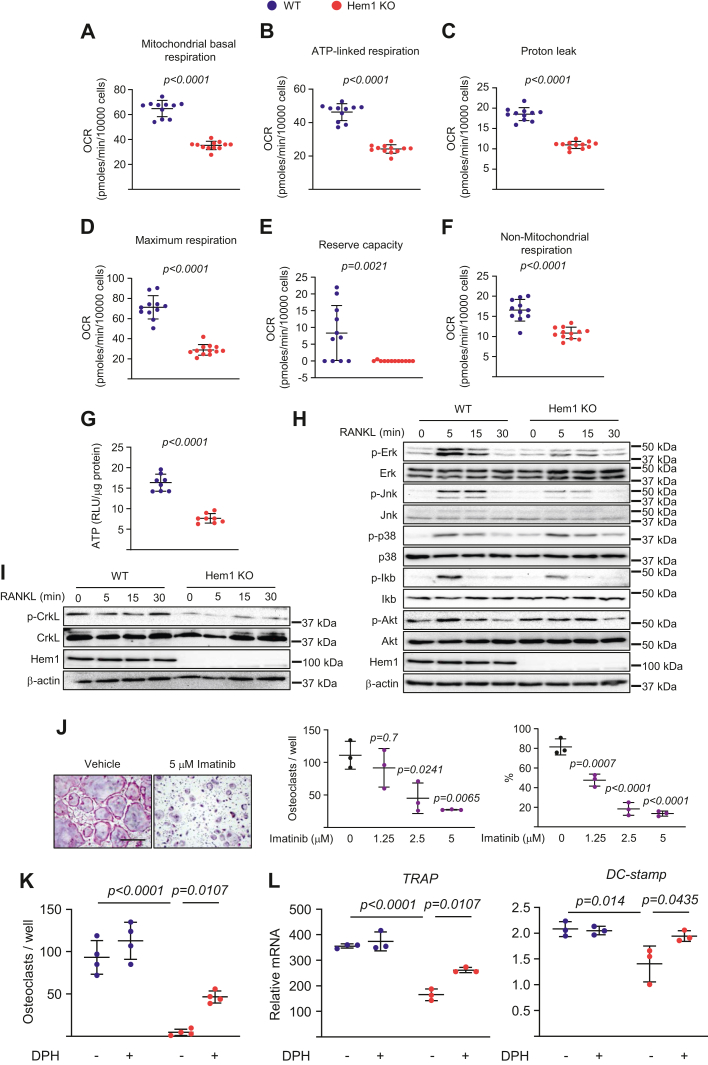
**Deletion of Hem1 attenuates respiration by suppressing c-Abl signaling.***A*–*F*, bone marrow macrophages from indicated genotypes were cultured with M-CSF and RANKL for 3 days. Different fractions of mitochondrial and nonmitochondrial respiration per 10,000 cells, in osteoclasts, were measured by Seahorse XF96 (n = 11–12 wells/group). *G*–*I*, bone marrow macrophages lacking Hem1 were cultured with M-CSF and RANKL for (*G*) 3 days or (*H* and *I*) indicated time points. *G*, ATP levels in osteoclasts (RLU, relative luminescence units; n = 4/group). *H* and *I*, protein levels (Western blot) in bone marrow macrophage cultures. To clarify Hem1 deletion in osteoclasts, the same images of Hem1 band are used. These are the same set of samples from the bone marrow macrophage cultures. *J*, osteoclasts developed in cultures of bone marrow macrophages from 6-month-old male C57BL/6 mice in the presence or the absence of imatinib (5 μM). Representative pictures (*left*), number (*middle*), and total area (*right*) of TRAP-positive multinucleated osteoclasts with more than ten nuclei (scale bar represents 500 μm) (triplicate cultures). *K* and *L*, bone marrow macrophages were isolated from 5.5-week-old male Hem1 knockout mice and wildtype littermates and were cultured with M-CSF and RANKL for (*K*) 5 or (*L*) 3 days in the presence or the absence of DPH (1 μM). *K*, number of TRAP-positive multinucleated osteoclasts generated from bone marrow macrophages. *L*, mRNA levels (quantitative PCR assay) of osteoclast markers during osteoclastogenesis. *Lines* and error bars represent mean ± SD. *p* Values were determined with (*A*–*G*) Student’s *t* test, (*J*) one-way ANOVA, or (*K* and *L*) two-way ANOVA. DPH, 5-(1, 3-diaryl-1H-pyrazol-4-yl); Hem1, hematopoietic protein 1; M-CSF, macrophage colony–stimulating factor; RANKL, receptor activator of nuclear factor-kappa B ligand; TRAP, tartrate-resistant acid phosphatase.

Activation of the Arp2/3 complex in yeast is indispensable for actin polymerization and cell migration and the accompanying enhanced mitochondrial function (17, 18). However, little is known about the role of Hem1 in regulation of mitochondrial function in mammalian cells. To examine this potential role of Hem1 in osteoclasts, we performed extracellular flux analysis, comparing osteoclasts from Hem1 knockout mice with those from wildtype mice. In osteoclasts from Hem1 knockout mice, mitochondrial respiration was significantly reduced (Fig. 5*A*), as were ATP-linked respiration, proton leakage, maximum respiration (the oxygen consumption rate when electron transport chain operates at maximum capacity), reserve respiratory capacity (flexibility of cells to increase oxygen consumption during increased energy demands), and nonmitochondrial respiration (Fig. 5, *B*–*F*). Consistent with the effects of Hem1 deletion on respiration, osteoclasts from Hem1 knockout mice had decreased ATP production in response to RANKL stimulation (Fig. 5*G*). Mitogen-activated protein kinase (MAPK), Akt, and both canonical and noncanonical NF-κB pathway are stimulated by RANKL and are essential for osteoclast survival and differentiation (19, 20, 21, 22, 23). We examined whether Hem1 alters RANKL-induced stimulation of these pathways. Cells from Hem1 knockout mice had reduced phosphorylation of Erk, Jnk, and IkB, whereas other signaling pathways, such as p38, Akt, (Fig. 5*H*), and RelB (Fig. S6) were not affected. Expression of NFATc1 and c-Fos, two essential transcription factors for early osteoclast differentiation, was not affected by Hem1 deletion (Fig. S6), suggesting that the inhibition of MAPK and canonical NF-κB pathway might contribute to the decreased mitochondrial activity seen in cells lacking Hem1.

We have shown that c-Abl, a tyrosine kinase that regulates actin polymerization and cell migration (24, 25, 26, 27), contributes to the impairment of hematopoietic stem cells in Hem1-deficient mice (13). Here, we examined whether c-Abl signaling also mediated the effects of Hem1 on osteoclast formation. Compared with osteoclasts from wildtype mice, those from Hem1 knockout mice had less phosphorylation of the c-Abl downstream target Crkl but no changes in total protein levels of Crkl (Fig. 5*I*). Addition of imatinib, a synthetic small-molecule inhibitor of c-Abl, to cultures of osteoclast progenitors from wildtype C57BL/6 mice resulted in decreased formation of osteoclasts (Fig. 5*J*), and most of these osteoclasts were small (Fig. 5*J*), reminiscent of osteoclasts from Hem1 knockout mice. We found no differences in cell survival and proliferation between vehicle and imatinib treatment (Fig. S7). Addition of 5-(1, 3-diaryl-1H-pyrazol-4-yl) hydantoin, a potent cell-permeable c-Abl activator, to cultures of osteoclast progenitors from Hem1 knockout mice significantly increased osteoclast formation and expression of *Dc-stamp* and *TRAP* (Fig. 5, *K* and *L*).

### Skeletal phenotype of Hem1 knockout mice is rescued by transplantation of bone marrow hematopoietic stem and progenitor cells from wildtype mice

**Figure 6:**
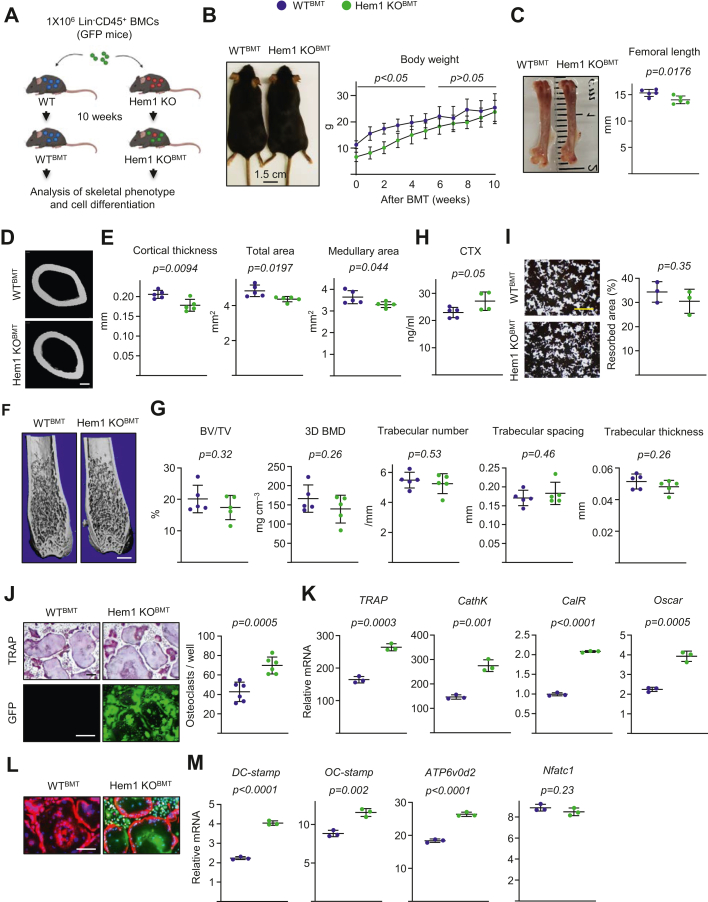
**Rescue of bone phenotype in Hem1-null mice by transplantation with wildtype hematopoietic cells.** Hematopoietic stem cells from GFP^+^Lin^−^CD45^+^ wildtype mice efficiently repopulated the hematopoietic system of nonablated Hem1-deficient mice after bone marrow cell transplantation. Femurs and bone marrow were analyzed 10 weeks after transplantation (n = 5). *A*, schematic model of bone marrow transplantation. *B*, representative images of whole body (*left*) and longitudinal weight measurement (*right*). *C*, representative images of whole femur (*left*) and length measurement in 13-week-old male recipient Hem1 knockout mice and wildtype littermates (*right*). *D*, representative images of cortical bone at midshaft (scale bar represents 100 μm). *E*, cortical thickness and cortical perimeters at the midshaft. *F*, representative images of trabecular bone (scale bar represents 1 mm) and (*G*) bone volume per tissue volume (BV/TV), bone mineral density (BMD), and microarchitecture of trabecular bone. *H*, serum concentration of a collagen degradation product (CTx) by ELISA. *I*, representative pictures (*left*) and resorbed areas (*right*) of Von Kossa-stained bone biomaterial surface (scale bar represents 500 μm) (triplicate cultures). *J*–*M*, bone marrow macrophages were isolated from 13-week-old recipient Hem1 knockout mice and wildtype littermates and cultured with M-CSF and RANKL for (*G*, *I*, and *J*) 5 days or (*K* and *M*) 3 days. *J*, representative pictures (*left*) and number (*right*) of TRAP-positive multinucleated osteoclasts with more than ten nuclei (scale bar represents 500 μm) (n = 6). *K*, mRNA levels (quantitative PCR assay) of osteoclast markers during osteoclastogenesis (triplicate cultures). *L*, representative pictures of DAPI (*blue*, nuclei) and phalloidin (*red*, actin rings) staining in osteoclast cultures (scale bar represents 500 μm). *M*, mRNA levels (quantitative PCR assay) of fusion markers during osteoclastogenesis (triplicate cultures). *Lines* and error bars represent mean ± SD. *p* Values were determined with Student’s *t* test. DAPI, 4′,6-diamidino-2-phenylindole; Hem1, hematopoietic protein 1; M-CSF, hematopoietic protein 1; RANKL, receptor activator of nuclear factor-kappa B ligand; TRAP, tartrate-resistant acid phosphatase.

Our previous studies showed that Hem1 knockout mice exhibited significant reductions in the numbers of total bone marrow nucleated cells, hematopoietic progenitor cells, hematopoietic stem cells, and cobblestone area-forming cells, which could be corrected by transplantation of normal wildtype bone marrow hematopoietic stem and progenitor cells (13). Therefore, we investigated whether transferring these cells could rescue the bone effects because of Hem1 deletion. We transplanted 1 × 10^6^ Lin^−^CD45^+^ bone marrow cells from normal C57BL/6-Tg (CD45.2/CAG-EGFP) mice into 3-week-old nonablated CD45.1 wildtype and Hem1 knockout mice (Fig. 6*A*). About 10 weeks after transplantation, most components of the hematopoietic system, including hematopoietic stem and progenitor cells, were comparable in wildtype and Hem1 knockout recipient mice (Fig. S8). In Hem1 knockout recipient mice, almost 90% of cells in the peripheral blood and bone marrow were derived from CD45.2/GFP donor cells; however, in wildtype recipient mice, no donor cells were detected. These results indicate that the transplanted normal hematopoietic cells reconstituted the hematopoietic system in Hem1 knockout recipient mice. Transplantation with normal hematopoietic cells also restored body size (Fig. 6*B*). Although not fully rescued, femoral length (Fig. 6*C*), cortical thickness, total area, and medullary area were much improved (Fig. 6, *D* and *E*) in transplanted Hem1 knockout mice than in nontransplanted Hem1 knockout mice (Fig. 1, *B* and *C*). Remarkably, trabecular bone volume and microarchitecture in the distal femur (Fig. 6, *F* and *G*) and serum levels of CTx in Hem1 knockout recipient mice were similar to wildtype (Fig. 6*H*). Consistently, *in vitro* bone-resorption ability was comparable for osteoclasts from both genotypes of recipient mice (Fig. 6*I*). In addition, osteoclast cultures from the bone marrow of transplanted Hem1 knockout mice had significantly increased osteoclast formation (Fig. 6*J*) and upregulated the expression of *TRAP*, *cathepsin K*, *calcitonin receptor*, and *Oscar* (Fig. 6*K*). Actin ring formation in osteoclasts from transplanted Hem1 knockout mice was comparable to that in wildtype mice, as was expression of *Dc-stamp*, *Oc-stamp*, *Atp6v0d2*, and *Nfatc1* (Fig. 6, *L* and *M*).

## Discussion

The pathways of osteoclastogenesis that regulate RANKL-induced osteoclast differentiation have been studied extensively, but less is known about osteoclast fusion, a unique feature of osteoclast maturation that is required for the proper bone resorption. Herein, we provide evidence that the hematopoietic-specific WAVE complex scaffold Hem1 is required for osteoclast fusion and bone-resorption activity and, thereby, contributes to the maintenance of bone mass in mice.

Our bone histomorphometric analysis demonstrated unexpectedly that the bone formation parameters, such as the osteoblast number and the serum concentration of osteocalcin, were significantly decreased or at least trended lower in Hem1 knockout mice, suggesting impaired bone formation. Thus, it could be concluded that the reduced osteoblast number and activity in Hem1 knockout mice would have resulted in low bone mass; however, a disproportionate effect on bone resorption could mask an effect of Hem1 on bone formation, resulting in high bone mass in Hem1 knockout mice. Similar findings have been reported in mice with loss-of-function mutations in proteins important for mitochondria in osteoclasts. For example, global deletion of PGC1β, a critical transcription factor for mitochondrial oxidative energy metabolism, decreases bone formation rate, mineralizing surface and osteoblast number but increases bone mass because of the impaired bone resorption (14).

Here, we showed that transplanting normal hematopoietic stem and progenitor cells into Hem1 knockout mice fully restored trabecular bone volume and microarchitecture in the distal femur as well as bone-resorption activity. These findings suggest that Hem1 is essential during growth for normal bone remodeling and for hematopoietic stem cell maintenance. Our previous studies showed that Hem1 knockout mice exhibit significant defects in the transition of hematopoiesis from the fetal liver to the bone marrow, which can be corrected by adoptive transfer of wildtype bone marrow hematopoietic stem and progenitor cells (13). This transfer also rescues growth retardation and premature death of Hem1 knockout mice. Thus, regeneration of the bone marrow microenvironment in Hem1 knockout mice has multiple other consequences besides restoration of osteoclast function.

In line with our findings, studies of short interfering RNA oligonucleotides in an osteoclast cell line revealed that the Arp2/3 complex regulates actin polymerization and promotes bone resorption (28), and other studies showed that the Arp2/3 complex locates in the core of the podosomes that make up actin rings, where it colocalizes with cortactin, a known regulator of Arp2/3 complex activity (29, 30). Interestingly, a recent study with mice lacking Hem1 have revealed that Hem1 is required for macrophage function (31). In yeast, the Arp2/3 complex promotes actin polymerization by regulating mitochondrial dynamics, but little is known about its role in mitochondria metabolism in mammalian cells (17, 18). Here, we showed that in osteoclasts, deletion of Hem1 decreased mitochondrial respiration. Overall, these findings support the notion that, Hem1, a component of the Rac-WAVE-Arp2/3 pathway, exerts effects in osteoclasts at least in part *via* an effect on mitochondrial function. Support for a link between Hem1, mitochondria, and bone resorption is provided by studies in which mitochondria activity has been attenuated in osteoclasts (14, 32, 33). These studies indicate that normal mitochondria metabolism is critical for osteoclast fusion and bone resorption. Nonetheless, future work is required to identify the range of target proteins and mitochondrial pathways that are responsible for the effects of Hem1 on osteoclast development.

A decrease in bone resorption was, most likely, responsible for the increase in trabecular bone mass in Hem1 knockout mice. Nonetheless, changes in mesenchymal lineage cell number, specifically CFU-F, CFU-AD, CFU-OB, and osteoblast were also noted. These changes are, most likely, indirect as the expression of Hem1 is very low in mesenchymal lineage cells (CD45^−^) from wildtype mice (Fig. S9). Strikingly, Hem1 knockout mice exhibited low numbers of osteoblast on trabecular bone surfaces, which could not be explained by changes in progenitor cell numbers indicated by CFU assays. One possible explanation for the decrease in osteoblasts could be the decrease in growth factors released from the matrix during bone resorption (7, 8). In view of the major decrease in osteoblast number in the Hem1 knockout mice, it is also possible that Hem1 plays a key role in supporting osteoblasts by stimulating osteoclasts to express coupling factors. Nonetheless, further biochemical and genetic studies are necessary to elucidate this possibility.

Bone homeostasis requires a bidirectional crosstalk between hematopoietic and mesenchymal lineage cells including osteoclasts, lymphocytes, osteoblasts, osteocytes, bone lining cells, adipocytes, as well as vascular endothelial cells by regulatory signaling pathways that tightly interact through complex autocrine/paracrine mechanisms (34). We counted CD45^−^Lin^−^CD31^−^Sca1^+^CD51^+^ mesenchymal stem cells in the bone marrow and found that the number of this cell population was higher in Hem1 knockout mice (Fig. S4), consistent with the observed increase in number of CFU. On the other hand, mRNA levels of osteoblast and adipocyte differentiation markers in fluorescence-activated cell sorted cells were reduced from that seen in wildtype littermate controls (Fig. S4). High-density stromal cell cultures also have decreased capacity to differentiate into osteoblasts or adipocytes (Fig. S4). The seemingly decreased capacity for differentiation in cells from Hem1 knockout mice might contribute to decrease in osteoblast number in bone. The changes in mesenchymal cells seen in Hem1 knockout mice are, most likely, secondary to the altered composition of the bone marrow microenvironment.

While our findings suggest that Hem1 in osteoclastic cells directly impact bone resorption, it is possible that changes in other hematopoietic cells contribute indirectly to the increased bone mass in Hem1 knockout mice. We found that Hem1 is essential for B cell, but not T cell, development (Fig. S3). Lymphocytes have been proposed to regulate bone mass *via* a variety of mechanisms (35, 36). Both *in vitro* and *in vivo* studies have suggested that B cells at several stages of differentiation, from pre-B cells to mature B lymphocytes, can support osteoclast differentiation (37, 38, 39, 40, 41). Therefore, the decrease in B-cell number might contribute to the low bone resorption in Hem1 knockout mice. Future studies are needed to further dissect the pathways *via* which Hem1 directly and indirectly impacts osteoclast formation.

We previously reported that the defects in hematopoietic cells in Hem1 knockout mice are associated with decreased c-Abl signaling. Here, we found that c-Abl signaling is also decreased in osteoclast progenitors. Inhibition of c-Abl in wildtype osteoclast progenitors caused defects in osteoclastogenesis similar to the ones caused by Hem1 deletion. These results suggest that c-Abl mediate the effects of Hem1 on osteoclast formation and function. Interestingly, c-Abl promotes mitochondrial dynamics in primary neurons *via* direct phosphorylation of target proteins such as Drp1 (42). This, together with our present findings that Hem1 stimulates mitochondria function, supports the notion that c-Abl also mediates the effects of Hem1 on mitochondria of osteoclasts.

RANK-proximal signaling pathways including MAPKs, Src, Akt, and NF-κB have been shown to be involved in osteoclast differentiation and function (19, 20, 21). Among these signaling pathways, ablation of Hem1 prevents the RANKL-induced NF-κB activation. Indeed, previous studies demonstrated that NF-κB plays a role in mitochondrial metabolism (43, 44), and this signaling is also essential for skeletal and mineral homeostasis (21). Therefore, future studies are needed to further dissect the pathways *via* which Hem1 regulates NF-κB signaling pathway in osteoclasts and whether this cascade impacts osteoclast mitochondria and function.

Based on the results of the present work, we propose that Hem1 directly regulates osteoclast fusion during bone development. In a separate line of work, we also showed that overexpression of Hem1 in cultured osteoclasts has enhanced actin ring formation and partially rescued the cytoskeletal defect in transferrin receptor 1 lacking osteoclasts (45). Further studies with models of cell lineage–specific deletion of Hem1 are needed to examine the role of Hem1 in adult bone both under physiological conditions and conditions of increased bone remodeling such as estrogen deficiency.

## Experimental procedures

### Mice

Hem1 knockout mice were bred in our facility from pairs of mice heterozygous for the Hem1 mutant allele (C57BL/6 background), as described previously (13). C57BL/6-Tg(CAG-EGFP) mice were purchased from Jackson Laboratories (Bar Harbor). All mice used in this study were housed under standard laboratory conditions with a 12 h dark, 12 h light cycle, a constant temperature of 23 °C, and humidity of 48%. A standard rodent diet (Envigo, Teklad 22/5) containing 22% protein, 1.13% calcium, and 0.94% phosphorus was provided to mice *ad libitum*. All mice were maintained at the University of Arkansas for Medical Sciences animal facility, which is accredited by the Association for the Assessment and Accreditation of Laboratory Animal Care International. Mice were randomly assigned to four or five mice per cage. All animal procedures were approved by the University of Arkansas for Medical Sciences Institutional Animal Care and Use Committee.

### Micro-CT analysis

Bone architecture was determined on dissected femurs that were cleaned of adherent soft tissues, fixed in Millonig’s phosphate buffer (Leica Microsystems), and gradually dehydrated in 100% ethanol. Bones were scanned with μCT40 (Scanco Medical) at high resolution for obtaining images and at medium resolution for making quantitative determinations as described previously (46). Specifically, scans were performed at medium resolution (12 μm isotropic voxel size) for quantitative determinations and integrated into 3-D voxel images (1024 × 1024 pixel matrices for each individual planar stack). To reduce signal noise, a Gaussian filter (sigma = 0.8, support = 1) was applied. Scano Eval Program version 6.0 (Scanco Medical) was used to measure bone volume. Scan settings were applied to X-ray tube potential (E = 55 kVp), X-ray intensity (I = 145 μA), and integration time (220 ms). Nomenclature conforms to recommendations of the American Society for Bone and Mineral Research (47). Trabecular bone measurements of the distal femur were made on 151 transverse slices that were taken from the epicondyles, extending toward the proximal end of the femur; cortical bone and primary spongiosa were manually excluded from the analysis. All trabecular analyses were performed on contours of every 10 to 20 cross-sectional images and were measured at a threshold of 220 mg/cm^3^. Trabecular architecture was determined using sphere filling distance–transformation indices without assumptions about the bone shape as a rod or a plate. Cortical dimensions were determined at the diaphysis (18 slices, midpoint of the bone length as determined in scout view) at a threshold of 200 mg/cm^3^.

### Bone histology

The terminology used in this report is that which is recommended by the Histomorphometry Nomenclature Committee of the American Society for Bone and Mineral Research (47). The femoral bones were fixed for 24 h in Millonig’s phosphate buffer, transferred to 100% ethanol, and embedded undecalcified in methyl methacrylate. For histomorphometric measurements, 5-μm-thick longitudinal sections were cut in the medial–lateral plane. To visualize osteoclasts, sections were stained with Naphthol AS-MX and Fast Red TR salt (Sigma–Aldrich) to detect TRAP activity. Sections also were stained with 0.3% toluidine blue in phosphate-buffered citrate, pH 3.7, to visualize osteoblasts, osteoid, and cement lines. Standard histomorphometric parameters including the number of TRAP-positive cells and osteoblasts on trabecular bone surface of longitudinal section were measured using an Olympus BX53 microscope and Olympus DP73 camera (Olympus America, Inc) interfaced with a digitizer tablet with Osteomeasure software, version 4.1.0.2 (OsteoMetrics, Inc). One section per sample was analyzed by a histopathologist blinded to the study groups.

### CTx and osteocalcin ELISA

Circulating CTx and osteocalcin in serum was measured using a mouse RatLaps (CTx-I) EIA kit (Immunodiagnostic Systems) and an Osteocalcin enzyme immunoassay kit (Thermo Fisher Scientific) according to the manufacturer’s directions. Blood was collected into 2.0 ml microcentrifuge tubes by retro-orbital bleeding. Blood was then kept on ice for 1 h and centrifuged at 10,000× rpm for 10 min to separate serum from cells.

### Osteoclast differentiation

Bone marrow macrophages were obtained as described previously (48). Briefly, bones (*i.e.*, femurs and tibias) were isolated and cleared of soft tissues. After red blood cells were removed using ACK buffer (0.01 mM EDTA, 0.011 M KHCO_3_, and 0.155 M NH4Cl, pH 7.3), the remaining cells were cultured with 10% fetal bovine serum (FBS), 100 units ml^−1^ of penicillin, and 100 μg ml^−1^ of streptomycin in the presence of 10 ng ml^−1^ M-CSF (R&D Systems) for 24 h. Nonadherent bone marrow cells were collected and cultured in petri dishes with 30 ng ml^−1^ M-CSF for 3 days to generate bone marrow macrophages; adherent bone marrow macrophages were harvested as osteoclast progenitors.

To generate preosteoclasts and osteoclasts, bone marrow macrophages were cultured in 6-well (for RT–PCR and Western blot analysis), 24-well (for resorption assays), or 48-well (for TRAP staining) plates in α-minimum essential complete medium with 30 ng ml^−1^ M-CSF and 30 ng ml^−1^ RANKL (R&D Systems) for 2 and 4 days, respectively. Imatinib (Selleckchem, catalog no.: S2475) and 5-(1, 3-diaryl-1H-pyrazol-4-yl) hydantoin (Sigma–Aldrich) were used in some of the experiments at the indicated concentrations. ATP levels were measured by a luciferin-luciferase–based assay using an ENLITEN ATP assay system bioluminescence detection kit (Promega) according to the manufacturer’s protocol, as previously described (49).

For coculture systems, bone marrow–derived stromal cells and bone marrow macrophages were seeded in 48-well plates with 1α,25(OH)_2_D_3_ (10^−8^ M; Sigma) and prostaglandin E_2_ (10^−8^M; Sigma) for 5 to 6 days in α--minimum essential complete medium.

To enumerate osteoclasts, cells were fixed with 10% neutral-buffered formalin for 10 min, permeabilized with 0.1% Triton X-100 in PBS for 5 min, and stained for TRAP with the Leukocyte Acid Phosphatase Assay Kit (Sigma–Aldrich), following the manufacturer’s instructions. Preosteoclasts were identified as round mononuclear TRAP-positive cells; osteoclasts were identified as multinucleated (>3 nuclei) TRAP-positive cells.

### Retroviral gene transduction

To prepare retroviral particles, Plat-E retroviral packaging cells were plated on a 10 mm culture dish and transfected with murine stem cell virus vectors encoding Hem1 using Lipofectamine 2000 (Invitrogen). After 3 days, the medium containing retroviruses was harvested and passed through a syringe filter (0.2 μm pore diameter). Bone marrow macrophages were infected with retroviruses for 8 h with 6 μg ml^−1^ polybrene (Sigma–Aldrich) in the presence of 30 ng ml^−1^ M-CSF. After washing with fresh medium, the cells were cultured for 2 days in the presence of 2 μg ml^−1^ puromycin (Sigma–Aldrich) with 30 ng ml^−1^ M-CSF. Puromycin-resistant bone marrow macrophages were studied.

### CFU assay

CFU-F, CFU-AD, and CFU-OB numbers were determined as previously described (50), 15% FBS, and 1 mM ascorbate-2-phosphate. Half of the medium was replaced every 5 days. CFU-Fs were enumerated at 10 days of culture after staining for AP, and CFU-OBs were enumerated at 25 days of culture after von Kossa staining. CFU-AD was enumerated at 6 days of culture in the presence of 1 μM rosiglitazone after Oil Red O staining.

### Bone resorption assay

Bone marrow macrophages were isolated as described previously and stimulated with RANKL to form osteoclasts on 24-well Osteo Assay Surface plates (Corning Life Sciences), which are coated with an inorganic bone biomaterial surface. Cells were removed with a 2% hypochlorite solution for 5 min, washed with distilled water, and dried at room temperature. For Von Kossa staining, wells were incubated for 20 min in darkness with 5% (w/v) aqueous silver nitrate solution (150 μl/well). Plates then were washed for 5 min with distilled water and incubated in darkness with 5% (w/v) sodium carbonate (150 μl/well) in 10% formalin solution. Wells were washed twice with PBS, rinsed with distilled water, and dried for 30 min at 50 °C. Each well was imaged with a microscope; the resorbed areas are white, and the unresorbed mineralized surface areas are black. Three wells were assessed for each group.

### Cellular bioenergetics

Application of extracellular flux analysis was performed as we previously described (49, 51). Bone marrow macrophages were plated and treated with 30 ng ml^−1^ M-CSF and 30 ng ml^−1^ RANKL for 3 days. The media in the wells were replaced with XF assay media, and the plate was kept in a non-CO_2_ incubator for 20 min at 37 °C. After recording three total cellular respiration measurements with the XF96 analyzer, 10 μg ml^−1^ oligomycin was added to inhibit mitochondrial ATP synthase and measure the decrease in the oxygen consumption rate that is linked to ATP turnover. To determine the maximal respiration potential of the cells, 10 μM carbonyl cyanide-*p*-trifluoromethoxyphenylhydrazone (FCCP; an uncoupler of oxidative phosphorylation) was used. The amount of nonmitochondrial oxygen consumption was determined by inhibiting the respiratory chain activity with an antimycin A and 10 μM rotenone cocktail. These data were used to calculate the mitochondrial basal respiration, ATP-linked respiration, reserve respiratory capacity, and proton leak.

### Hematopoietic cell transplantation

Bone marrow mononuclear cells were isolated from 8-week-old GFP mice and resuspended at the concentration of 10^7^ cells ml^−1^. Cells were incubated with purified lineage antibodies (CD11b, Gr1, CD3e, B220, Ter119, and BD) for 30 min at 4 °C and then washed twice with 2% FBS/Hanks' balanced salt solution. The labeled mature cells were depleted by incubation with goat antirat immunoglobulin G magnetic beads (Thermo Fisher Scientific) in a magnetic field. The isolated Lin^−^ cells were washed and incubated with APC-Cy7-conjugated antimouse CD45 antibody. Lin^−^CD45^+^ cells were sorted with an Aria II cell sorter (BD Biosciences). About 1 × 10^6^ GFP^+^Lin^−^CD45^+^ cells were retro-orbitally transplanted into a 3-week-old Hem1 knockout mouse or wildtype littermate control. Body weights were measured every week after transplantation. Mice were analyzed at 10 weeks after transplantation. Femurs and tibias were isolated, and bone marrow macrophages and bone marrow mononuclear cells were collected for subsequent analysis.

### RNA isolation and quantitative RT–PCR analysis

Total RNA was purified from cultured bone marrow–derived macrophages or bone marrow stromal cells using TRIzol reagent (Thermo Fisher Scientific) according to the manufacturer's directions. From 2 μg of total RNA extract, complementary DNA (cDNA) was obtained with the High-Capacity cDNA Archive Kit (Applied Biosystems) according to the manufacturer’s instructions. TaqMan quantitative real-time PCR was performed with the following primers from Applied Biosystems: TRAP (Mn00475698_m1), Ctsk (Mm00484039_m1), Calcr (Mm00432271_m1), Oscar (Mm00558665_m1), DC-Stamp (Mm04209236_m1), Atp6v0d2 (Mm01222963_m1), Oc-Stamp (Mm00512445_m1), and NFATc1 (Mm00479445_m1). To measure Hem1 expression, the SYBR assay kit was used (Applied Biosystems). Briefly, 1 μl cDNA was mixed with 7.5 μl SYBR Green PCR Master Mix and 0.2 μl of primers. Samples were then added into 6.30 μl of water (for a total volume of 15 μl). Quantitative PCR conditions were as follows: 95 °C for 10 min, 40 cycles of 95 °C for 15 s and 60 °C for 1 min, 95 °C for 15 min, 60 °C for 60 min, and 95 °C for 15 min. All reactions were run in triplicate, and target gene expression was calculated by normalizing to the housekeeping gene ribosomal protein S2 (Mm00475528_m1) or hypoxanthine phosphoribosyltransferase (forward: 5′-AGCAGTACAGCCCCAAAATGGTTA-3′ and reverse: 5′-TCAAGGGCATATCCAACAACAAAC-3) with the ΔCt method (52).

### Western blot analysis

Cells were lysed with a buffer containing 20 mM Tris–HCl, 150 mM NaCl, 1% Triton X-100, protease inhibitor mixture, and phosphatase inhibitor cocktail (Sigma–Aldrich). After incubation on ice for 30 min, the cell lysates were centrifuged at 13,200 rpm for 15 min at 4 °C. Protein concentrations of cell lysate samples were determined with the DC Protein Assay kit (Bio-Rad). The extracted proteins (40 μg per sample) were separated electrophoretically on 8 to 10% SDS-PAGE gels and then transferred onto polyvinylidene difluoride membranes. The membranes were blocked in 5% fat-free milk/Tris-buffered saline for 90 min and incubated with each primary antibody, followed by secondary antibodies conjugated to horseradish peroxidase. Monoclonal antibodies against NFATc1 (Santa Cruz Biotechnology; catalog no.: sc-7294, 1/500 dilution), CrkL (Cell Signaling Technology; catalog no.: 3182, 1/1000 dilution), p-Erk (Santa Cruz Biotechnology; catalog no.: sc-7383, 1/500 dilution), Erk (Santa Cruz Biotechnology; catalog no.: sc-94, 1/500 dilution), p-Jnk (Cell Signaling; catalog no.: 9255, 1/1000 dilution), Jnk (Santa Cruz Biotechnology; catalog no.: sc-1648, 1/500 dilution), p-p38 (Cell Signaling; catalog no.: 9215, 1/1000 dilution), p-IκB (Cell Signaling, catalog no.: 9246, 1/1000 dilution), p-Akt (Cell Signaling; catalog no.: 4058, 1/1000 dilution), and β-actin (Santa Cruz Biotechnology, catalog no.: sc-81178, 1/2000 dilution) were used to detect their corresponding protein levels. Also, we used polyclonal antibodies for Hem1 (Novus Biologicals; catalog no.: NBP2-13643, 1/1000 dilution), c-Fos (Santa Cruz Biotechnology; catalog no.: sc-7202, 1/500 dilution), p38 (Cell Signaling; catalog no.: 9212, 1/1000 dilution), IκB (Santa Cruz Biotechnology; catalog no.: sc-847, 1/500 dilution), Akt (Cell Signaling; catalog no.: 9272, 1/1000 dilution), and p-CrkL (Cell Signaling Technology; catalog no.: 3181, 1/1000 dilution) to analyze their protein levels. Band intensities in the autoradiograms were quantified with a VersaDoc imaging system (Bio-Rad). All Western blot analyses were performed at least two times using bone marrow macrophages pooled from 3 to 4 mice from each group.

### Actin ring staining

Bone marrow macrophages were cultured in 6-well plates with 30 ng ml^−1^ M-CSF and 30 ng ml^−1^ RANKL to form osteoclasts. The cells were fixed with 4% paraformaldehyde in PBS for 15 min and blocked in 1% BSA in PBS for 1 h. Actin fibers and nuclei were stained with phalloidin–rhodamine conjugate (Invitrogen) and 4′,6-diamidino-2-phenylindole (Vector Laboratories), respectively. The stained cells were viewed and photographed with an Axioplan research microscope (Carl Zeiss, Inc) equipped with a 100-W mercury light source. Images were captured with a Dage CCD100 integrating camera (Dage-MTI) and a Flashpoint 128 capture board (Integral Technologies). The captured images were processed with Image Pro Plus software (Media Cybernetics).

### Statistical analyses

All *in vitro* assays were repeated at least once. The data were analyzed by ANOVA or Student’s *t* test (independent samples, two-sided) with GraphPad Prism 9 from GraphPad Software, after determining that the data were normally distributed and exhibited equivalent variances. In the event that ANOVA justified post hoc comparisons between group means, these were conducted with Tukey’s multiple-comparisons test.

### Study approval

The Institutional Animal Care and Use Committees of the University of Arkansas for Medical Sciences and the Central Arkansas Veterans Healthcare System reviewed and approved all studies involving mice.

## Data availability

All the data indicated in this study are available upon request by contract from the corresponding author.

## Supporting information

This article contains supporting information.

## Conflict of interest

The authors declare that they have no conflicts of interest with the contents of this article.
