# Supplementary material for: Hematopoietic cytoplasmic adaptor protein Hem1 promotes osteoclast fusion and bone resorption in mice
Source: J Biol Chem. 2022 Dec 24;299(2):102841. doi: 10.1016/j.jbc.2022.102841 (PMC9867982; doi:10.1016/j.jbc.2022.102841)
Supplement: Supplemental Figures S1–S9 [file mmc1.pdf]

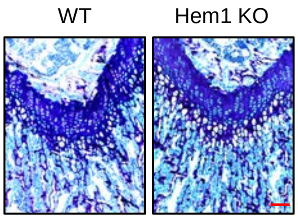

Supporting Fig. 1: Histological sections of distal femurs stained with toluidine blue (scale bar: 100 μm).

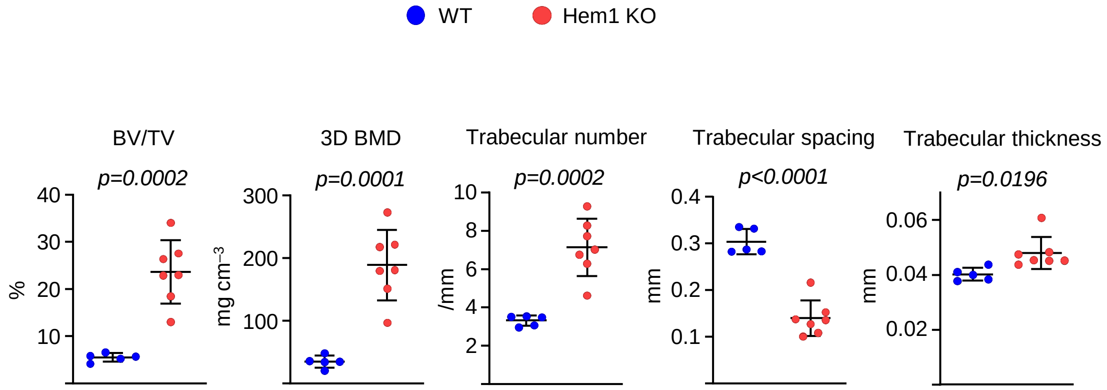

Supporting Fig. 2: Bone volume per tissue volume (BV/TV), bone mineral density (BMD), and microarchitecture of trabecular bone in distal femur of 5.5-week-old female mice (n = 5–7), determined with micro-CT. Lines and error bars represent mean ± SD. *P* values determined with Student's *t*-test.

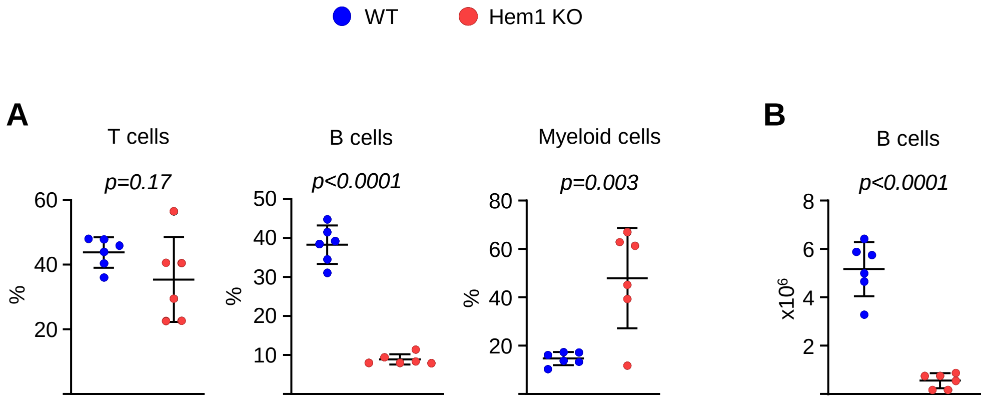

Supporting Fig. 3: (**A**) Percentage of T cells, B cells and myeloid cells, and (**B**) absolute number of B cells in bone marrow analyzed by flow cytometry (*n* = 6). P values determined with Student's t-test.

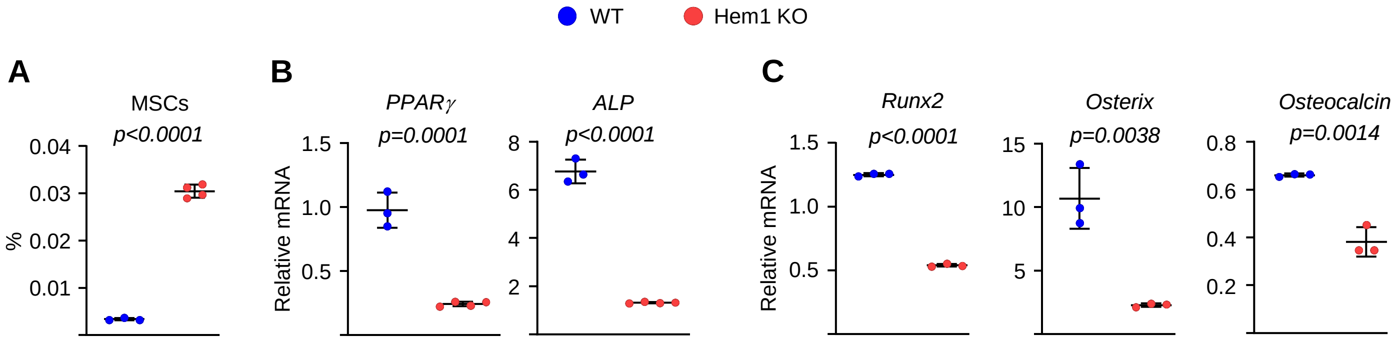

Supporting Fig. 4: (**A**) Percentage of $CD45^{-}Lin^{-}CD31^{-}Sca1^{+}CD51^{+}$ mesenchymal stem cells (MSCs) in bone marrow analyzed by flow cytometry (n = 3–4). (**B**) PPARγ and ALP mRNA levels in the sorted MSCs measured by qPCR. (**C**) Bone marrow stromal cells were isolated from 5.5-week-old male Hem1 knockout mice and wild-type littermates cultured with ascorbate (50 µg/ml) and β-glycerophosphate (10 mM) for 5 days. Osteoblast marker levels in pre-osteoblasts measured by qPCR. P values determined with Student's t-test.

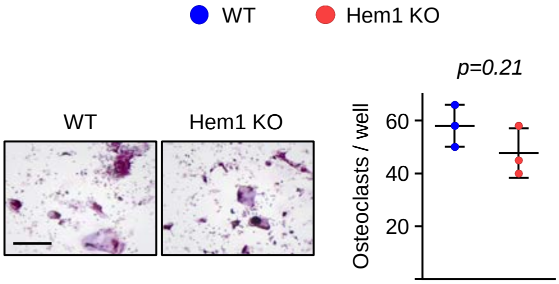

Supporting Fig. 5: Bone marrow macrophages from 3-month-old wild-type C57BL/6J female mice were co-cultured for 7 days with stromal cells from 5.5-week-old male Hem1 knockout mice and wild-type littermates. Representative pictures (left) and number (right) of TRAP-positive multinucleated cells (scale bar: 500 µm). P values determined with Student's t-test.

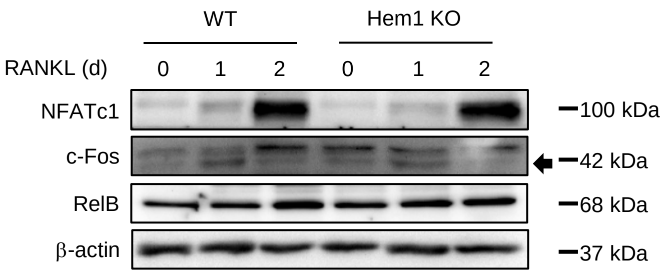

Supporting Fig. 6: Bone marrow macrophages from indicated genotypes were cultured with M-CSF and RANKL for indicated time points. Protein levels (Western blot) in bone marrow macrophage cultures.

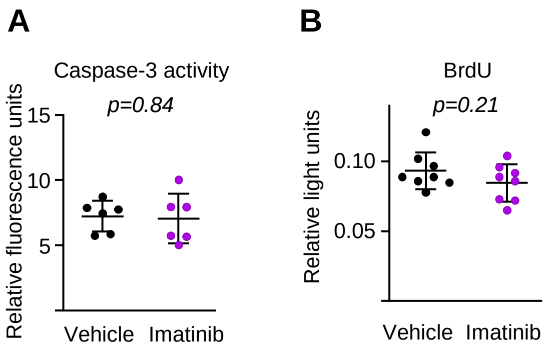

Supporting Fig. 7: Osteoclasts developed in cultures of bone marrow macrophages from 6-month-old male C57BL/6 mice in the presence or absence of imatinib (5 μM) for last 24 hours of culture period. (A) Apoptosis by caspase-3 activity. (B) Proliferation by BrdU labeling. P values determined with Student's t-test.

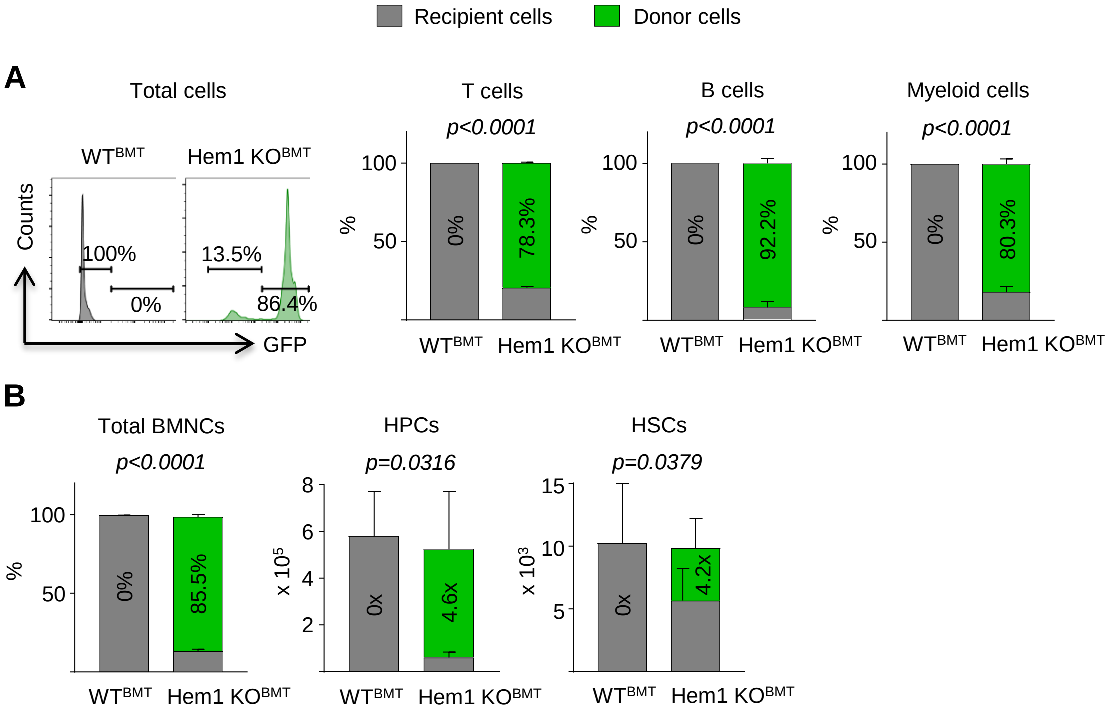

Supporting Fig. 8: Percentage of hematopoietic lineages in (A) peripheral blood and (B) bone marrow analyzed by flow cytometry (n = 3–4). P values determined with Student's t-test.

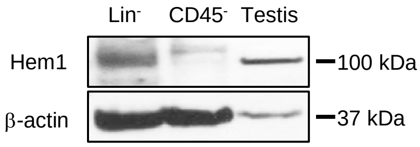

Supporting Fig. 9: Hem1 Protein levels (Western blot) in the isolated cells from bone marrow. Testis tissues were used as a control.
